# Surviving the storm: exploring gender-based burnout in Lebanon during the COVID-19 pandemic

**DOI:** 10.3389/fpubh.2025.1464268

**Published:** 2025-02-17

**Authors:** Aniella Abi-Gerges, Hani Dimassi, Myriam Boueri, Edwin Feghali, Melissa Bou Malham, Marie Josee Salem, Ranime Harb, Elma Nassar, Jana Mourad, Myriam Sfeir, Lamis R. Karaoui

**Affiliations:** ^1^Gilbert and Rose-Marie Chagoury School of Medicine, Lebanese American University, Byblos, Lebanon; ^2^Department of Pharmaceutical Sciences, School of Pharmacy, Lebanese American University, Byblos, Lebanon; ^3^Graduate Studies & Research Office, Lebanese American University, Byblos, Lebanon; ^4^Public Economics Unit, University of Kassel, Faculty of Economics and Management, Kassel, Germany; ^5^Arab Institute for Women, Lebanese American University, Beirut, Lebanon; ^6^Department of Pharmacy Practice, School of Pharmacy, Lebanese American University, Byblos, Lebanon

**Keywords:** burnout, gender, mental health, Lebanon, good health and well-being

## Abstract

**Background:**

COVID-19 has worsened burnout, marked by exhaustion, cynicism, and reduced professional efficacy. In Lebanon, economic collapse, political instability, the Beirut Port explosion, and social unrest have compounded this, with limited gender-specific data. This study evaluates burnout levels in Lebanese women and men during COVID-19, exploring gender differences and related factors, including burnout mitigation strategies.

**Methods:**

A cross-sectional survey included 423 adult participants aged 18 or older from Lebanon, recruited via online snowball sampling during July 2021 to August 2022. Utilizing the Maslach Burnout Inventory-General Survey (MBI-GS), participants reported exhaustion (≥ 12), cynicism (≥ 11), and low professional efficacy (≤ 21). Burnout was defined as exhaustion with either cynicism or low efficacy. The survey gathered demographic, family, and professional data, stressors, and burnout mitigation strategies. Analyses were gender-stratified, using descriptive statistics and Pearson’s chi-squared test. Bivariate associations between burnout indicators and sample characteristics were tested using Pearson’s chi-square. Odds ratios (OR) and adjusted ORs for burnout were estimated via logistic regressions.

**Results:**

Lebanese women experienced higher rates of burnout compared to men during the pandemic, with significant differences observed in emotional exhaustion (*p* = 0.006). Factors associated with burnout varied between genders, with women more likely to exhibit exhaustion when residing in the Beqaa, Mount or North Lebanon governorates, being single, having children aged 10–15 years, and most interestingly when lacking support from their boss/institution. Stressors such as the Lebanese economic crisis and the Beirut Port Explosion were significantly linked to burnout in both genders, with the economic crisis particularly associated with higher levels of exhaustion in men (*p* = 0.011) and cynicism in both genders (*p* = 0.001 for men, *p* = 0.039 for women). Coping strategies, including COVID-19 precautions, social activities, and religious practices, were effective in reducing burnout among both women and men who experienced burnout (*p* = 0.039 and 0.03, respectively).

**Conclusion:**

The study contributes to public health efforts, emphasizing the importance of recognizing gender dimensions in addressing burnout during the pandemic, designing targeted interventions and fostering supportive, inclusive environments for collective resilience namely for women at the workplace.

## Introduction

Burnout is an occupational phenomenon characterized by a persistent reaction to ongoing emotional and interpersonal workplace stressors. It is characterized by three fundamental components: exhaustion, cynicism, and low professional efficiency (1). The first aspect, exhaustion, pertains to the experience of stress, particularly persistent fatigue arising from overwhelming work requirements. The second aspect, depersonalization or cynicism, involves an indifferent or detached approach toward work and colleagues, resulting in a diminished interest in one’s tasks and a sense that the significance of work has decreased. Lastly, a deficiency in professional efficacy signifies diminished feelings of effectiveness, achievement, and success, both within one’s role and the broader organizational context. Emotional exhaustion and cynicism are both categorized as types of occupational strains and viewed as the core components of burnout (1).

### Literature review

The advent of the global coronavirus disease 2019 (COVID-19) pandemic has impacted all aspects of human life, particularly the mental health and overall well-being of the population through diverse challenges such as isolation and loneliness, job loss, financial instability, as well as illness, death, and grief. Individuals were forced into confinement and social distancing, transitioned to the “new normal” of work, and tried to adapt to unfamiliar circumstances that may have led to emotional, physical, and mental exhaustion, and ultimately burnout. The risk of burnout was elevated as a result of several factors such as the decline in social support, heightened workloads, and rapidly evolving public health directives (2). More so, Pietrabissa et al. showed that social isolation and the associated emotional and psychological challenges have been significant sources of burnout during the pandemic (3). For instance, resignations hit a record rate and have been attributed to people making changes to their work-life balance and exiting the workforce at a faster pace and in greater numbers due to various factors, such as seeking better childcare options and tending to family caregiving responsibilities (4). Parents faced significant challenges in the care and management of their children during the pandemic and are recognized as a population at risk for developing burnout. Despite studies indicating that the health crisis has not led to a surge in cases of parental burnout, numerous parents did express feelings of exhaustion (5).

While burnout has traditionally been considered gender-neutral, recent evidence suggests that the pandemic’s impact may have been disproportionately borne by women due to their dual burden of professional obligations and increased caregiving duties at home (6, 7). Disasters often amplify existing inequalities, leading to poorer mental health, greater unemployment, and social isolation among women and girls (8).

Lebanon is a small coastal eastern Mediterranean country, uniquely blending both eastern and western cultures, has been hit by multiple layers of crises. Lebanon’s economic collapse, compounded by political instability and social unrest, presents a compelling case for studying burnout. The collapse of the banking system, currency devaluation, hyperinflation, and widespread poverty have led to job losses, salary cuts, and financial insecurity, exacerbating mental health issues. In fact, the nation’s real gross domestic product (GDP) per capita declined for the eleventh successive year in 2021, accompanied by a substantial devaluation of the exchange rate. Thus, Lebanon, previously classified as an “Upper-Middle Income” country for nearly a quarter of a century, was reassigned to the “Lower-Middle Income” category (9). Amid the COVID-19 pandemic and the economic turmoil, the devastating explosion of the Beirut port on August 4, 2020 resulted in more than 200 fatalities, left more than 6,500 injured and rendered 300,000 others homeless (10). Furthermore, the strain on the healthcare system and breakdown of social support networks intensified feelings of helplessness and isolation, further contributing to burnout among individuals and communities. These snowballing crises have led to a deteriorating quality of life, and collective suffering and uncertainty in the Lebanese population. Burnout in Lebanon has been mostly assessed in the healthcare professions of medicine (11–14), pharmacy (15), under stable non-crisis conditions and during the COVID-19 pandemic (16). To the best of our knowledge, limited data is published about gender differences and burnout in Lebanon, and more specifically during a time of crisis such as COVID-19. This study is pivotal in filling the gap in understanding burnout in Lebanon, particularly among women and men during times of compounded crises. By focusing on gender differences, it provides valuable insights into how societal roles, economic pressures, and crisis conditions shape the burnout experience. Such findings could inform targeted interventions and policies to mitigate burnout in Lebanon and similar settings facing multifaceted challenges. In light of the myriad adverse consequences experienced by women and men during the pandemic, this study aims to assess the level of burnout among Lebanese women and men during the COVID-19 pandemic and identify gender differences and other related factors including mitigation strategies adopted during the lockdown. It examines the levels of burnout among Lebanese women and men during the COVID-19 pandemic, and how gender differences and related factors contribute to burnout.

The study offers new insights into the interplay between gender, crisis conditions, and burnout, emphasizing the importance of context-specific strategies to address occupational stress. By exploring mitigation strategies adopted during the lockdown, this research contributes to a framework for effective crisis adaptation and mental health resilience, particularly in fragile socio-economic environments.

## Materials and methods

### Study population

A cross-sectional snowball convenience sample of 423 adult women and men, 18 years or older, who witnessed the COVID-19 pandemic, was recruited from the general Lebanese population through an anonymous online survey that required 15–20 min to be completed. The online survey, which was created using Google Forms platform, was shared through social media channels such as Facebook, LinkedIn, Twitter, and WhatsApp. Data collection took place upon the participants’ consent during the period between July 2021 and August 2022.

### Study design

The online self-administered Google questionnaire consisted of a total of 51 close-ended questions distributed as follows: 11 questions were about the respondents’ sociodemographic characteristics and their personal lifestyle; eight questions tackled the traveling history of participants, their experience in sharing household responsibilities, contribution to the online education of their children and the impact of the latter on their level of stress and satisfaction; 14 questions assessed the participants’ work style during COVID-19 (office or remote work; schedule, etc.), their access to resources, productivity, and satisfaction rate; 16 questions were adopted from the validated Maslach Burnout Inventory™—General Survey (MBI-GS) (17) to assess the effect of COVID-19 pandemic on burnout in Lebanese women and men; and the last 2 questions identified the stress factors and the mitigation strategies adopted by the respondents during COVID-19. The beginning of the questionnaire included an informed consent form that clearly stated the purpose of the research and emphasizes the importance of voluntary participation, confidentiality, and anonymity of the information. The survey questions were accessible exclusively to individuals who consented to participate in this study. The contact information of the principal investigator/corresponding author of this study and the Institutional Review Board (IRB) committee at the Lebanese American University (LAU) were also included should the respondents wish to ask questions or withdraw from the study. The questionnaire was pilot-tested and edited before its online administration. The English questionnaire was translated to Arabic and back translated to English for consistency and validity by a certified translator. The consenting participants were given the choice to use either the English or Arabic questionnaire. Reminder emails were also sent to participants in a timely manner. By choosing to complete the survey, participants provided their informed consent to participate in the study. The study was approved by the IRB committee at LAU and granted exempt status under the code number LAU.SOP.LK1.21/Jun/2021.

### Statistical analysis

Data collected was coded and entered into SPSS V29 for analysis. The 16 items of the MBI-GS questionnaire were scored on a scale of 0 to 6, with 7 indicating inapplicable. MBI-GS questionnaire included three subscales: (1) exhaustion was measured with five items with a possible score of 0–30, where a score of ≥12 indicates the presence of exhaustion; (2) cynicism was measured with five items with a possible score of 0–30, where a score of ≥11 indicates presence of cynicism; (3) professional efficacy was measured with six items with a possible score of 0–36, where a score of ≤21 indicates low professional efficacy (15). Burnout was considered present if exhaustion was identified coupled with either cynicism and/or low professional efficacy. All analyses were stratified by gender. Descriptive statistics were summarized using counts and percentages and compared using the Pearson’s chi-squared test. A bivariate association between exhaustion, cynicism, and low professional efficacy and sample characteristics were tested using Pearson’s chi-square. Odds ratio (OR) and adjusted OR for burnout with all sample characteristics were estimated using simple and multivariable logistic regressions. Confidence intervals and *p* values were also reported. Statistical significance was determined at the 5% level.

## Results

### Characteristics of the surveyed population

Four hundred and twenty-three adults consented to participate in the study and their characteristics are presented in Table 1. The average age of the surveyed population was 37.9 ± 11.9 years, with the average age of men being 35.7 ± 13.3 years (*n* = 120), and the average age of women being 38.8 ± 11.1 years (*n* = 303). Most of the respondents, irrespective of their gender, had either completed a bachelor’s degree or pursued postgraduate studies (e.g., MS, MD, and PhD) and were mainly from the Beirut and Mount Lebanon governorates. More males (17.5%) were living alone during the lockdown compared to females (10.2%; *p* = 0.040). Around 38% of women and men had help at home provided by either the family (36.7% help for men vs. 21.8% for women; *p* = 0.002) or the helper (6.7% for men vs. 20.8% help for women vs.; *p* < 0.001). The respondent’s marital status was split between single (59.2% men vs. 36.3% women) and married (40.8% men vs. 63.7% women; *p* < 0.001). More than 85% of both men and women reported having kids of different ages and less than 15% were expecting a baby.

**Table 1:** Characteristics of the surveyed population by gender (*n* = 423).

	Female		Male		*p*-value
	N	%	N	%	
Age					
18–39 years old	154	50.8%	75	62.5%	
≥ 40 years old	149	49.2%	45	37.5%	0.030
Level of education					
Up to high school	23	7.6%	13	10.8%	
Technical/vocational school	9	3.0%	10	8.3%	
Bachelor’s degree (e.g., BA, BS)	132	43.9%	48	40.0%	
Higher studies (e.g., MS, MD, PhD)	137	45.5%	49	40.8%	0.067
Lebanese governorate					
Beirut	75	24.8%	22	18.3%	
Beqaa	42	13.9%	11	9.2%	
Mount Lebanon	142	46.9%	69	57.5%	
North Lebanon	23	7.6%	11	9.2%	
South Lebanon	21	6.9%	7	5.8%	0.246
Living alone during the lockdown					
No	272	89.8%	99	82.5%	
Yes	31	10.2%	21	17.5%	0.040
Help at home					
No	187	61.7%	73	60.8%	
Yes	116	38.3%	47	39.2%	0.866
Provider of help at home					
Family	66	21.8%	44	36.7%	0.002
Helper	63	20.8%	8	6.7%	<0.001
Others	5	1.7%	2	1.7%	0.990
Marital status					
Single	110	36.3%	71	59.2%	
Married	193	63.7%	49	40.8%	<0.001
Having kids					
No	25	12.1%	9	15.0%	
Yes	182	87.9%	51	85.0%	0.550
Number of kids					
1	33	18.1%	15	29.4%	
2	81	44.5%	14	27.5%	
3	51	28.0%	16	31.4%	
≥ 4	17	9.3%	6	11.8%	0.289
Kids age range					
0–5 years	45	14.9%	17	14.2%	0.858
5–10 years	52	17.2%	16	13.3%	0.334
10–15 years	45	14.9%	9	7.5%	0.041
15–20 years	45	14.9%	4	3.3%	0.001
20–25 years	17	5.6%	13	10.8%	0.059
25 years +	50	16.5%	13	10.8%	0.140
Expectancy of a baby					
No	181	87.4%	51	85.0%	
Yes	26	12.6%	9	15.0%	0.622

### Personal lifestyle changes during COVID-19 lockdown and pandemic

Changes in the personal lifestyle of participants were ascertained by a set of eight questions displayed in Table 2. More women (61.5%) were involved in the online learning of their children as compared to men (41%; *p* = 0.009) and reported higher levels of stress (11.8% of men vs. 34.9% of women expressed the highest level of stress; *p* = 0.022). No differences were reported in the satisfaction level of both men and women participants with respect to the online learning of their children. More than 83% of the respondents stated that their spouse was residing in Lebanon during the lockdown; however only around half of the participants of both genders shared responsibilities equally at home. No differences between men and women were reported regarding the traveling history and its frequency during the lockdown. Finally, only 2.6% of women and 1.7% of men reported experiencing domestic violence (*p* = 0.552).

**Table 2:** Family and professional duties by gender.

	Female		Male		*p*-value
	N	%	N	%	
Family duties					
Involvement in children online learning					
No	70	38.5%	30	58.8%	
Yes	112	61.5%	21	41.2%	0.009
Level of stress experienced due to the online learning of children					
1 (Lowest Level)	20	13.4%	11	32.4%	
2	11	7.4%	4	11.8%	
3	36	24.2%	9	26.5%	
4	30	20.1%	6	17.6%	
5 (Highest Level)	52	34.9%	4	11.8%	0.022
Satisfaction with respect to children’s online learning					
1 (Lowest Level)	25	16.9%	7	20.6%	
2	24	16.2%	5	14.7%	
3	60	40.5%	13	38.2%	
4	24	16.2%	3	8.8%	
5 (Highest Level)	15	10.1%	6	17.6%	0.616
Residency of spouse in Lebanon					
No	27	13.0%	10	16.7%	
Yes	180	87.0%	50	83.3%	0.474
Sharing responsibilities equally at home					
No	103	49.8%	26	43.3%	
Yes	104	50.2%	34	56.7%	0.381
Traveling abroad					
No	276	91.1%	105	87.5%	
Yes	27	8.9%	15	12.5%	0.266
Frequency of travel					
Frequent	11	42.3%	7	46.7%	
Not Frequent	15	57.7%	8	53.3%	0.786
Experiencing domestic violence					
No	295	97.4%	118	98.3%	
Yes	8	2.6%	2	1.7%	0.552
Professional duties					
Working during lockdown					
No	102	33.8%	39	32.5%	
Yes	200	66.2%	81	67.5%	0.802
Occupation during lockdown					
Blue Collar	18	9.0%	15	18.5%	
White Collar	155	77.5%	51	63.0%	
Healthcare	27	13.5%	15	18.5%	0.029
Type of work contract during lockdown					
Part Time	51	25.5%	18	22.2%	
Full Time	149	74.5%	63	77.8%	0.563
Work situation during lockdown					
Working at the office	40	20.0%	28	34.6%	
Working remotely from home	103	51.5%	24	29.6%	
Working remotely and at the office (hybrid)	57	28.5%	29	35.8%	0.002
COVID-19 restrictions at workplace					
No	14	14.4%	9	15.8%	
Yes	83	85.6%	48	84.2%	0.820
Compliance with professional deadlines					
No	30	15.0%	12	14.8%	
Yes	170	85.0%	69	85.2%	0.969
Support from institution/boss					
No	55	27.5%	26	32.1%	
Yes	145	72.5%	55	67.9%	0.441
Similar working schedule while working from home					
No	117	60.6%	53	67.9%	
Yes	76	39.4%	25	32.1%	0.259
Working hours from home					
< 2 Hours	8	7.0%	10	20.4%	
2–4 Hours	31	27.0%	11	22.4%	
5–7 Hours	32	27.8%	10	20.4%	
8–10 Hours	24	20.9%	3	6.1%	
> 10 Hours	20	17.4%	15	30.6%	0.007
Working on weekends from home					
No	74	37.9%	31	39.7%	
Yes	121	62.1%	47	60.3%	0.783
Easy access to technology (laptop, desktop, smartphone, other)					
No	22	10.9%	15	18.5%	
Yes	179	89.1%	66	81.5%	0.088
Easy access to Internet					
No	51	25.4%	18	22.2%	
Yes	150	74.6%	63	77.8%	0.578
Satisfaction with professional productivity					
1	6	3.0%	7	8.6%	
2	31	15.4%	13	16.0%	
3	72	35.8%	25	30.9%	
4	65	32.3%	22	27.2%	
5	27	13.4%	14	17.3%	0.238
More time spent on task					
Strongly disagree	12	6.0%	7	8.6%	
Disagree	32	15.9%	13	16.0%	
Neutral	36	17.9%	24	29.6%	
Agree	88	43.8%	26	32.1%	
Strongly agree	33	16.4%	11	13.6%	0.159

### Professional status and productivity during COVID-19 lockdown and pandemic

Table 2 also shows the work style of both men and women during COVID-19 as well as their productivity, and satisfaction rate. More than 66% of men and women respondents were working during the COVID-19 lockdown on a full-time basis. While most of them were white collar workers (77.5% of women vs. 63.0% of men), a minority was reported to be working in the healthcare sector (18.5% of men vs. 13.5% of women; *p* = 0.029). The work situation during the lockdown was split between working at the office (34.6% of men vs. 20.0% of women) where the majority reported that the COVID-19 safety measures were taken seriously at their workplace, remotely from home (29.6% of men vs. 51.5% of women) and hybrid (35.8% of men vs. 28.5% of women; *p* = 0.002). When asked about whether they were able to meet the professional deadlines, 85% of the respondents agreed, irrespective of their gender, and the majority confirmed that they were getting the needed support from their boss/institution (67.9% of men vs. 72.5% of women, *p* = 0.441). Furthermore, 67.9% of men and 60.6% of women reported not being able to adopt the same office working schedule (Monday to Friday from 8:00 a.m. until 5:00 p.m.) while working from home with 30.6% of men compared to 17.4% of women working more than 10 h daily (*p* = 0.007) and around 60% of both men and women working during weekends. More than 70% of the participants, irrespective of their gender, had easy access to technology and internet. Around 44% of the participants, irrespective of their gender, reported high satisfaction with respect to their overall professional productivity during the pandemic/lockdown (average satisfaction score in both men and women was 2.2 ± 1.8) although 45.7% of men and 60.2% of women felt that they spent more time to complete the same task that they used to complete before the lockdown.

### Effect of COVID-19 pandemic on Lebanese women and men’s burnout

**Figure 1:**
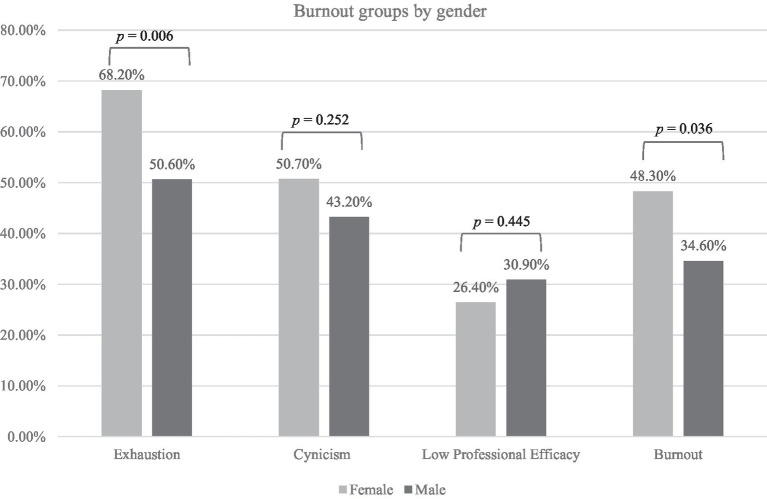
Bar chart representing exhaustion, cynicism, low professional efficacy, and burnout by gender.

**Figure 2:**
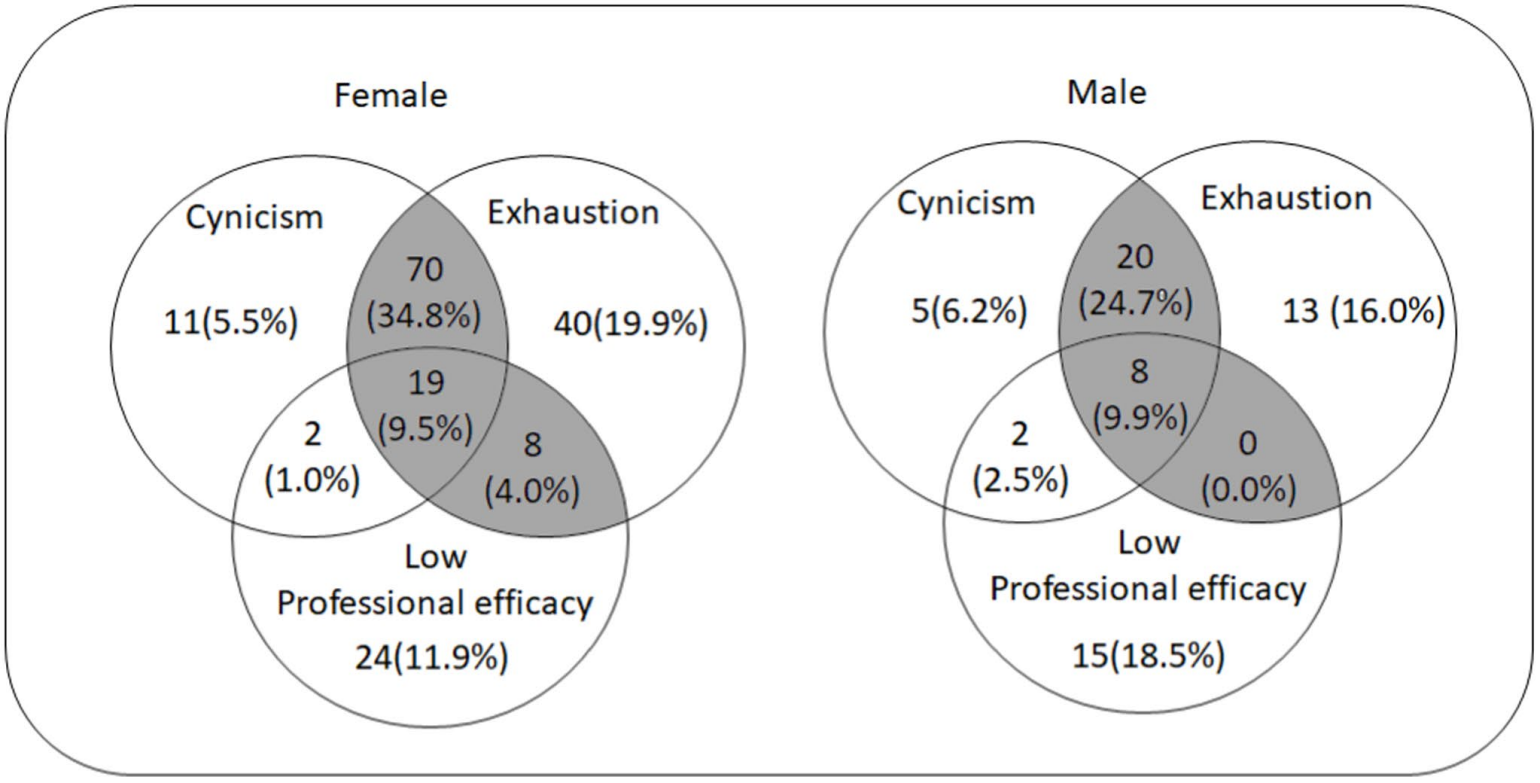
Venn diagram depicting exhaustion, cynicism and low professional efficacy for female and male gender.

The effect of COVID-19 pandemic on Lebanese women and men’s burnout was ascertained by a set of 16 questions that were adopted from the validated MBI-GS (Tables 3, 4; Figures 1, 2).

**Table 3:** Factors associated with exhaustion, cynicism, and low professional efficacy stratified by gender.

	Exhaustion						Cynicism						Low professional efficacy					
	Female			Male			Female			Male			Female			Male		
	N	%	*P*	N	%	*P*	N	%	*P*	N	%	*P*	N	%	*P*	N	%	*P*
Age																		
18–39 years old	79	70.5%		21	45.7%		65	58.0%		18	39.1%		24	21.4%		17	37.0%	
≥ 40 years old	58	65.2%	0.417	20	57.1%	0.306	37	41.6%	0.020	17	48.6%	0.395	29	32.6%	0.075	8	22.9%	0.174
Level of education																		
Up to high school	8	88.9%		8	88.9%		7	77.8%		6	66.7%		4	44.4%		3	33.3%	
Technical/vocational school	2	66.7%		2	33.3%		0	0.0%		3	50.0%		0	0.0%		2	33.3%	
Bachelor’s degree (BA, BS)	60	75.9%		13	44.8%		46	58.2%		9	31.0%		18	22.8%		7	24.1%	
Higher studies (MS, MD, PhD)	67	61.5%	0.099	18	48.6%	0.092	49	45.0%	0.030	17	45.9%	0.262	30	27.5%	0.362	13	35.1%	0.808
Lebanese governorate																		
Beirut	30	54.5%		4	40.0%		27	49.1%		3	30.0%		16	29.1%		3	30.0%	
Beqaa	18	81.8%		2	22.2%		15	68.2%		2	22.2%		7	31.8%		4	44.4%	
Mount Lebanon	73	73.0%		29	60.4%		53	53.0%		24	50.0%		20	20.0%		12	25.0%	
North Lebanon	12	80.0%		2	25.0%		6	40.0%		3	37.5%		5	33.3%		5	62.5%	
South Lebanon	4	44.4%	0.027	4	66.7%	0.095	1	11.1%	0.054	3	50.0%	0.486	5	55.6%	0.138	1	16.7%	0.209
Living alone during the lockdown																		
No	121	68.0%		35	51.5%		91	51.1%		30	44.1%		47	26.4%		18	26.5%	
Yes	16	69.6%	0.878	6	46.2%	0.725	11	47.8%	0.766	5	38.5%	0.706	6	26.1%	0.974	7	53.8%	0.050
Help at home																		
No	83	66.9%		29	55.8%		66	53.2%		25	48.1%		30	24.2%		15	28.8%	
Yes	54	70.1%	0.637	12	41.4%	0.214	36	46.8%	0.372	10	34.5%	0.236	23	29.9%	0.375	10	34.5%	0.599
Provider of help																		
Family	31	73.8%	0.377	12	44.4%	0.432	20	47.6%	0.649	10	37.0%	0.428	12	28.6%	0.716	9	33.3%	0.734
Helper	26	60.5%	0.222	0	0.0%	0.038	17	39.5%	0.097	0	0.0%	0.074	14	32.6%	0.299	2	50.0%	0.395
Others	3	100.0%	0.233	1	50.0%	0.986	1	33.3%	0.543	0	0.0%	0.212	0	0.0%	0.296	1	50.0%	0.553
Marital status																		
Single	57	80.3%		21	51.2%		45	63.4%		20	48.8%		13	18.3%		15	36.6%	
Married	80	61.5%	0.006	20	50.0%	0.913	57	43.8%	0.008	15	37.5%	0.306	40	30.8%	0.055	10	25.0%	0.259
Having kids																		
No	13	61.9%		3	33.3%		11	52.4%		4	44.4%		7	33.3%		4	44.4%	
Yes	73	61.3%	0.961	21	52.5%	0.299	53	44.5%	0.506	14	35.0%	0.595	36	30.3%	0.778	12	30.0%	0.404
Number of kids																		
1	17	60.7%		6	42.9%		13	46.4%		5	35.7%		9	32.1%		11	78.6%	
2	35	62.5%		7	63.6%		23	41.1%		2	18.2%		14	25.0%		0	0.0%	
3	17	65.4%		6	60.0%		13	50.0%		3	30.0%		9	34.6%		1	10.0%	
≥ 4	4	44.4%	0.729	2	40.0%	0.491	4	44.4%	0.890	4	80.0%	0.188	4	44.4%	0.599	0	0.0%	<0.001
Kids age range																		
0–5 years	20	57.1%	0.124	6	40.0%	0.362	17	48.6%	0.777	3	20.0%	0.044	9	25.7%	0.923	4	26.7%	0.697
5–10 years	26	70.3%	0.760	7	46.7%	0.735	19	51.4%	0.935	3	20.0%	0.044	7	18.9%	0.255	5	33.3%	0.819
10–15 years	12	48.0%	0.021	2	28.6%	0.222	11	44.0%	0.471	1	14.3%	0.106	11	44.0%	0.033	1	14.3%	0.320
15–20 years	13	54.2%	0.117	2	66.7%	0.571	12	50.0%	0.938	1	33.3%	0.725	10	41.7%	0.070	0	0.0%	0.238
20–25 years	2	40.0%	0.171	5	55.6%	0.753	4	80.0%	0.185	5	55.6%	0.428	2	40.0%	0.484	2	22.2%	0.552
25 years +	19	65.5%	0.741	4	66.7%	0.414	12	41.4%	0.275	4	66.7%	0.228	8	27.6%	0.872	2	33.3%	0.892
Involvement in children online learning																		
No	25	55.6%		12	52.2%		18	40.0%		11	47.8%		15	33.3%		8	34.8%	
Yes	48	64.9%	0.312	9	52.9%	0.962	35	47.3%	0.437	3	17.6%	0.048	21	28.4%	0.568	4	23.5%	0.443
Level of stress experienced due to the online learning of children																		
1 (Lowest Level)	7	63.6%		5	62.5%		5	45.5%		4	50.0%		1	9.1%		1	12.5%	
2	2	25.0%		4	100.0%		3	37.5%		2	50.0%		2	25.0%		1	25.0%	
3	14	56.0%		3	50.0%		7	28.0%		3	50.0%		9	36.0%		1	16.7%	
4	14	73.7%		1	20.0%		9	47.4%		0	0.0%		4	21.1%		1	20.0%	
5 (Highest Level)	26	74.3%	0.079	3	75.0%	0.159	21	60.0%	0.180	1	25.0%	0.344	13	37.1%	0.353	1	25.0%	0.979
Satisfaction with respect to children’s online learning																		
1 (Lowest Level)	9	64.3%		3	50.0%		7	50.0%		2	33.3%		2	14.3%		1	16.7%	
2	8	50.0%		1	20.0%		8	50.0%		1	20.0%		4	25.0%		0	0.0%	
3	28	68.3%		6	75.0%		17	41.5%		4	50.0%		16	39.0%		3	37.5%	
4	13	72.2%		2	66.7%		6	33.3%		0	0.0%		5	27.8%		1	33.3%	
5 (Highest Level)	5	55.6%	0.644	4	80.0%	0.269	7	77.8%	0.253	2	40.0%	0.558	2	22.2%	0.441	0	0.0%	0.327
Expectancy of a baby																		
No	77	64.7%		23	56.1%		57	47.9%		17	41.5%		37	31.1%		13	31.7%	
Yes	9	42.9%	0.058	1	12.5%	0.024	7	33.3%	0.217	1	12.5%	0.120	6	28.6%	0.817	3	37.5%	0.749
Residency of spouse in Lebanon																		
No	8	47.1%		4	44.4%		9	52.9%		4	44.4%		4	23.5%		6	66.7%	
Yes	78	63.4%	0.194	20	50.0%	0.763	55	44.7%	0.523	14	35.0%	0.595	39	31.7%	0.493	10	25.0%	0.016
Sharing responsibilities equally at home																		
No	42	65.6%		13	56.5%		34	53.1%		6	26.1%		18	28.1%		8	34.8%	
Yes	44	57.9%	0.349	11	42.3%	0.321	30	39.5%	0.106	12	46.2%	0.146	25	32.9%	0.542	8	30.8%	0.765
Traveling abroad																		
No	121	68.0%		35	51.5%		91	51.1%		30	44.1%		42	23.6%		23	33.8%	
Yes	16	69.6%	0.878	6	46.2%	0.725	11	47.8%	0.766	5	38.5%	0.706	11	47.8%	0.013	2	15.4%	0.187
Frequency of travel																		
Frequent	7	70.0%		1	20.0%		5	50.0%		1	20.0%		5	50.0%		2	40.0%	
Not frequent	9	69.2%	0.968	5	62.5%	0.135	6	46.2%	0.855	4	50.0%	0.279	6	46.2%	0.855	0	0.0%	0.052
Experiencing domestic violence																		
No	134	67.7%		40	50.0%		101	51.0%		34	42.5%		51	25.8%		25	31.3%	
Yes	3	100.0%	0.233	1	100.0%	0.320	1	33.3%	0.543	1	100.0%	0.249	2	66.7%	0.110	0	0.0%	0.501
Working during lockdown																		
No	0	0.0%		0	0.0%		0	0.0%		0	0.0%		0	0.0%		0	0.0%	
Yes	136	68.0%		41	50.6%		102	51.0%		35	43.2%		53	26.5%		25	30.9%	
Occupation during lockdown																		
Blue collar	12	66.7%		9	60.0%		8	44.4%		8	53.3%		3	16.7%		3	20.0%	
White collar	105	68.2%		26	51.0%		79	51.3%		22	43.1%		40	26.0%		17	33.3%	
Health	18	66.7%	0.982	6	40.0%	0.547	14	51.9%	0.853	5	33.3%	0.543	9	33.3%	0.458	5	33.3%	0.601
Type of work contract during lockdown																		
Part time	30	58.8%		9	50.0%		27	52.9%		11	61.1%		15	29.4%		8	44.4%	
Full time	106	71.1%	0.104	32	50.8%	0.953	75	50.3%	0.748	24	38.1%	0.082	38	25.5%	0.585	17	27.0%	0.157
Work situation during lockdown																		
Working at the office	31	77.5%		15	53.6%		25	62.5%		12	42.9%		15	37.5%		7	25.0%	
Working remotely from home	68	66.0%		14	58.3%		47	45.6%		13	54.2%		26	25.2%		6	25.0%	
Working remotely and at the office (hybrid)	37	64.9%	0.351	12	41.4%	0.436	30	52.6%	0.186	10	34.5%	0.354	12	21.1%	0.179	12	41.4%	0.310
COVID-19 restrictions at workplace																		
No	11	78.6%		4	44.4%		9	64.3%		5	55.6%		4	28.6%		5	55.6%	
Yes	57	68.7%	0.454	23	47.9%	0.848	46	55.4%	0.536	17	35.4%	0.255	23	27.7%	0.947	14	29.2%	0.123
Compliance with professional deadlines																		
No	24	80.0%		7	58.3%		23	76.7%		6	50.0%		9	30.0%		5	41.7%	
Yes	112	65.9%	0.126	34	49.3%	0.562	79	46.5%	0.002	29	42.0%	0.607	44	25.9%	0.638	20	29.0%	0.380
Support from institution/boss																		
No	48	87.3%		17	65.4%		43	78.2%		16	61.5%		16	29.1%		6	23.1%	
Yes	88	60.7%	<0.001	24	43.6%	0.068	59	40.7%	<0.001	19	34.5%	0.022	37	25.5%	0.609	19	34.5%	0.297
Similar working schedule while working from home																		
No	82	70.1%		25	47.2%		61	52.1%		23	43.4%		29	24.8%		19	35.8%	
Yes	50	65.8%	0.531	15	60.0%	0.290	37	48.7%	0.639	10	40.0%	0.777	21	27.6%	0.659	4	16.0%	0.073
Working hours from home																		
< 2 Hours	6	75.0%		4	40.0%		6	75.0%		4	40.0%		2	25.0%		6	60.0%	
2–4 Hours	21	67.7%		5	45.5%		13	41.9%		6	54.5%		12	38.7%		3	27.3%	
5–7 Hours	22	68.8%		5	50.0%		18	56.3%		4	40.0%		7	21.9%		4	40.0%	
8–10 Hours	21	87.5%		3	100.0%		16	66.7%		3	100.0%		4	16.7%		1	33.3%	
> 10 Hours	12	60.0%	0.322	5	33.3%	0.320	7	35.0%	0.109	4	26.7%	0.179	3	15.0%	0.258	4	26.7%	0.483
Working on weekends from home																		
No	48	64.9%		17	54.8%		43	58.1%		16	51.6%		21	28.4%		9	29.0%	
Yes	85	70.2%	0.433	22	46.8%	0.488	56	46.3%	0.109	18	38.3%	0.246	29	24.0%	0.494	15	31.9%	0.787
Easy access to technology (laptop, desktop, smartphone, other)																		
No	16	72.7%		9	60.0%		15	68.2%		10	66.7%		7	31.8%		5	33.3%	
Yes	121	67.6%	0.626	32	48.5%	0.421	87	48.6%	0.083	25	37.9%	0.042	46	25.7%	0.539	20	30.3%	0.819
Easy access to Internet																		
No	35	68.6%		10	55.6%		27	52.9%		11	61.1%		16	31.4%		7	38.9%	
Yes	102	68.0%	0.934	31	49.2%	0.635	75	50.0%	0.717	24	38.1%	0.082	37	24.7%	0.348	18	28.6%	0.403
Satisfaction with professional productivity																		
1	5	83.3%		5	71.4%		5	83.3%		5	71.4%		3	50.0%		4	57.1%	
2	24	77.4%		6	46.2%		21	67.7%		6	46.2%		10	32.3%		5	38.5%	
3	47	65.3%		11	44.0%		40	55.6%		13	52.0%		20	27.8%		10	40.0%	
4	44	67.7%		8	36.4%		26	40.0%		6	27.3%		17	26.2%		5	22.7%	
5	17	63.0%	0.648	11	78.6%	0.095	10	37.0%	0.019	5	35.7%	0.223	3	11.1%	0.232	1	7.1%	0.093
More time spent on task																		
Strongly disagree	7	58.3%		4	57.1%		7	58.3%		2	28.6%		1	8.3%		3	42.9%	
Disagree	21	65.6%		5	38.5%		12	37.5%		6	46.2%		6	18.8%		2	15.4%	
Neutral	25	69.4%		9	37.5%		17	47.2%		7	29.2%		13	36.1%		11	45.8%	
Agree	56	63.6%		15	57.7%		42	47.7%		13	50.0%		26	29.5%		6	23.1%	
Strongly agree	28	84.8%	0.221	8	72.7%	0.266	24	72.7%	0.052	7	63.6%	0.293	7	21.2%	0.228	3	27.3%	0.262

**Table 4:** Association between burnout and participants characteristics stratified by gender.

	Burnout							
	Female				Male			
	Bivariate Unadjusted OR (95% CI)	*p*-value	Multivariate Adjusted* OR (95% CI)	*p*-value	Unadjusted OR (95% CI)	*p*-value	Adjusted* OR (95% CI)	*p*-value
Age								
18–39 years old	Ref				Ref			
≥ 40 years old		0.049			1.221 (0.486–3.071)	0.671		
Level of education								
Up to high school	Ref		Ref		Ref			
Technical/vocational school	^	–	Ref		0.250 (0.028–2.237)	0.215		
Bachelor’s degree (e.g., BA, BS)	0.157 (0.019–1.317)	0.088	Ref		0.190 (0.038–0.950)	0.043		
Higher studies (e.g., MS, MD, PhD)	0.088 (0.011–0.728)	0.024	0.550 (0.300–1.000)	0.050	0.240 (0.051–1.128)	0.071		
Help at home								
No	Ref				Ref		Ref	
Yes	0.833 (0.471–1.474)	0.531			0.356 (0.124–1.020)	0.054	0.220 (0.050–0.900)	0.035
Marital status								
Single	Ref				Ref			
Married	0.554 (0.308–0.994)	0.048			0.535 (0.211–1.359)	0.189		
Involvement in children online learning								
No	Ref				Ref		Ref	
Yes	1.627 (0.759–3.485)	0.211			0.173 (0.032–0.939)	0.042	0.020 (0.001–0.250)	0.003
Work situation during lockdown								
Working at the office	Ref				Ref			
Working remotely from home	0.466 (0.220–0.985)	0.045			1.080 (0.348–3.349)	0.894		
Working remotely and at the office (hybrid)	0.540 (0.237–1.232)	0.143			0.810 (0.269–2.441)	0.708		
Compliance with professional deadlines								
No	Ref				Ref			
Yes	0.347 (0.150–0.801)	0.013			0.468 (0.135–1.617)	0.230	*	
Support from institution/boss								
No	Ref		Ref		Ref		Ref	
Yes	0.189 (0.093–0.383)	<0.001	0.210 (0.100–0.430)	<0.001	0.227 (0.084–0.615)	0.004	0.190 (0.040–0.920)	0.039
Satisfaction with professional productivity								
1	Ref				Ref		Ref	
2	0.420 (0.043–4.087)	0.455			0.250 (0.034–1.819)	0.171	0.030 (0.001–0.720)	0.030
3	0.189 (0.021–1.701)	0.137			0.225 (0.036–1.405)	0.111	0.050 (0.002–1.110)	0.058
4	0.142 (0.016–1.286)	0.083			0.118 (0.017–0.802)	0.029	0.010 (0.000–0.360)	0.012
5	0.100 (0.010–0.989)	0.049			0.160 (0.021–1.192)	0.074	0.020 (0.001–0.660)	0.027
COVID coping strategies								
No	Ref		Ref		Ref		Ref	
Yes	0.460 (0.227–0.934)	0.032	0.440 (0.200–0.960)	0.039	0.455 (0.176–1.182)	0.106	0.243 (0.070–0.870)	0.030

*Adjusted to covariates that remained statistically significant in the multivariable logistic regression, with the exception of “compliance with professional deadlines” that remain the model for male due to impact of significance of “support from institution/boss.” Ref: stands for reference group in reading the OR, in the multivariate logistic regression for female the education variable was recategorized to compare higher education group with all others. ^model did not produce OR due to 0 cell.

Figure 1 shows the proportion of men and women respondents who experienced burnout with an analysis of its three components: emotional exhaustion, cynicism, and diminished professional efficacy. More women participants reported burnout compared to men (34.6% of men vs. 48.3% of women; *p* = 0.036) with a specifically higher percentage experiencing exhaustion (50.6% of men vs. 68.2% of women; *p* = 0.006) while there was no difference in cynicism (43.2% of men vs. 50.7% of women; *p* = 0.252) and low professional efficacy (30.9% of men vs. 26.4% of women; *p* = 0.445) between both groups.

Table 3 shows factors associated with burnout components (exhaustion, cynicism, and low professional efficacy) stratified by gender. Among women, being a resident of Beqaa, Mount and North Lebanon governorates, being single, having children aged 10–15 years, and lacking boss/institution support were associated with a higher likelihood of individuals exhibiting exhaustion. The percentage of women expressing cynicism differed significantly according to age, level of education, marital status, compliance with professional deadlines, support from the boss/institution and satisfaction with professional productivity. The percentage experiencing low professional efficacy differed significantly according to age of kids and travel. Among men, the proportion of individuals exhibiting exhaustion differed significantly according to the nature of help provided at home and whether they were expecting a baby. The percentage of individuals expressing cynicism significantly correlated with the age of kids, involvement in the online learning of children, support from the boss/institution, and access to technology. The percentage of men experiencing low professional efficacy correlated significantly with whether they were living alone during lockdown, residency of their spouse in Lebanon, and the number of their kids.

Figure 2 shows a Venn diagram that illustrates the percentage of men and women expressing exhaustion, cynicism, and low professional efficacy. Burnout was considered present if exhaustion was experienced with either cynicism and/or low professional efficacy. Among women, 34.8% experienced exhaustion and cynicism, 9.5% showed exhaustion, cynicism, and low professional efficacy, whereas 4.0% expressed exhaustion and low professional efficacy. In men, 24.7% experienced exhaustion and cynicism while 9.9% showed exhaustion, cynicism, and low professional efficacy. No man expressed exhaustion and low professional efficacy together.

OR and adjusted OR for burnout with all sample characteristics were estimated using simple and multivariable logistic regressions (Table 4).

Bivariate analysis showed that a lower odd of women experiencing burnout significantly correlated with age above 40 years, university level of education, being married, working remotely from home, compliance with professional deadlines, being provided with the needed support from the boss/institution, being satisfied with their professional productivity and adopting COVID-19 coping strategies. After adjustment, a decrease in the prevalence of burnout in women was still significantly associated with post graduate studies (odds ratio and confidence interval of 0.55 [0.30–1.00]; *p* = 0.05), being provided with the needed support from the boss/institution (odds ratio and confidence interval of 0.21 [0.10–0.43]; *p* < 0.001) and adopting COVID-19 coping strategies (odds ratio and confidence interval of 0.44 [0.0–0.96]; *p* = 0.039).

Based on the bivariate analysis, the prevalence of burnout was lower among men who had a university degree, were involved in their children online learning, received support from the boss/institution and were satisfied with their professional productivity. After adjustment, a decrease in the prevalence of burnout in men significantly associated with help at home (odds ratio and confidence interval of 0.22 [0.05–0.90]; *p* = 0.035), involvement in the online learning of their children (odds ratio and confidence interval of 0.02 [0.001–0.25]; *p* = 0.003), support provided by the boss/institution (odds ratio and confidence interval of 0.19 [0.04–0.92]; *p* = 0.039), satisfaction with professional productivity (odds ratio and confidence interval of 0.02 [0.001–0.66]; *p* = 0.027), and adopting COVID-19 coping strategies (odds ratio and confidence interval of 0.243 [0.07–0.87]; *p* = 0.030).

### Stress factors and coping strategies adopted during COVID-19 pandemic

The surveyed population was asked to identify the stressors that they experienced during the COVID-19 pandemic. The different stressors were cross-tabulated with the 16 questions of the MBI-GS to evaluate the association between burnout and the stress factors in both men and women (data not shown). Around half of the surveyed women (52.1%) reported being stressed by the COVID-19 pandemic, Lebanon’s economic crisis and the 2020 Beirut Port Explosion. Among men, the highest number expressing burnout reported the Lebanese economic crisis as the main stressor (40.6%; *p* = 0.006), followed by the COVID-19 pandemic (37.5%) then the 2020 Beirut Port Explosion (34.0%). Among the three stressors, the Lebanese economic crisis was significantly linked with a higher percentage of exhaustion (56.5% vs. 16.7%, *p* = 0.011) in men and cynicism in both men (50.7% vs. 0%; *p* = 0.001) and women (52.7% vs. 23.1%; *p* = 0.039).

Different coping strategies were employed by the respondents during the COVID-19 pandemic, including COVID-19 precautions (hygiene, social distancing andwearing masks), entertainment and social activities (chatting with family and friends, browsing social media, watching television, reading, cooking, online shopping, sleeping) and religious practices (praying). Coping strategies were helpful in both women and men who had burnout (*p* = 0.039 and 0.03 respectively).

## Discussion

The current study explores gender-based burnout predictors in Lebanon during the COVID-19 pandemic. Despite common features, differences in burnout experiences between Lebanese women and men were observed. More women reported burnout with specifically a higher prevalence of exhaustion compared to men while there were no gender-related differences in cynicism and low professional efficacy. The COVID-19 pandemic has not only presented unprecedented challenges to global health but has also significantly impacted various aspects of our lives, including work and mental well-being. As we navigated through these uncertain times, it was crucial to examine how different demographic groups were affected, and one such dimension is gender. Burnout, a state of chronic physical and emotional exhaustion often related to work-related stressors, has been on the rise, and understanding its manifestations in women and men can provide valuable insights into developing targeted interventions and support systems. Employers, policymakers, and individuals must address the unique challenges faced by women and men, providing resources to mitigate burnout and promote mental well-being. While complete elimination of burnout is unrealistic, organizations can foster growth, empowerment, shared governance, enhanced communication, and gender equity to improve job satisfaction (18). Creating supportive, inclusive workplaces, particularly for women, is essential for resilience and warrants further study.

The higher prevalence of exhaustion in women is consistent with the results of a comprehensive meta-analysis of 180 studies, which found that women are more likely to report exhaustion-type burnout while men are more likely to report depersonalization-type burnout (19). The gender-specific patterns of burnout observed among the study participants during the COVID-19 pandemic could be attributed to family dynamics, societal, occupational, or individual factors. Women and men respondents reported similarities with respect to help provided at home and equal distribution of household responsibilities. However, with the closure of schools, Lebanese women were more involved in their children’s online learning compared to men and consequently reported higher levels of stress. This is supported by emerging research suggesting that women have provided more childcare than men during the COVID-19 pandemic even while continuing to work (20, 21). Furthermore, numerous studies have documented the pervasive impact of stress on the family system, with parenting stress affecting parent–child interactions, and concurrently children’s stress impacting parents’ stress, sense of parenting competence, and overall well-being (22–24). With the increased caregiving demands for children, women who traditionally shouldered diverse roles at home and in the workplace faced an exacerbated burden during the pandemic in balancing professional responsibilities with caregiving duties (25). This was problematic both for women who had the privilege to work from home and even more so for women who were essential employees. For those that can work from home, the expectations on women to manage domestic family needs while also managing full-time careers and work may be unreasonable. Importantly, our findings showed that women were mainly working remotely from home, compared to the majority of men who were working either at the office or in a hybrid mode. With remote work blurring the boundaries between professional and personal life, the expectation to excel in both arenas has intensified contributing to heightened stress levels (26).

Despite meeting professional deadlines and getting the needed support from their boss/institution, the majority of women and men respondents reported not being able to adopt the same office-working schedule while working from home, spending more time to complete the same task they used to complete before the lockdown and were even compelled to work during weekends. Consequently, less than half of the participants, irrespective of their gender, reported high satisfaction with respect to their overall professional productivity during the pandemic/lockdown.

Importantly, certain factors had varying impacts on burnout and its components for women and men. One of the most prominent findings of our study is that the support from boss/institution and adoption of COVID-19 coping strategies decreased the prevalence of burnout in both women and men. This was in agreement with previous studies that focused on both the human aspect of work and the value of human resources that play a primordial role in meeting the worker’s needs (27) and eventually the job expectations. A workplace that fosters empowerment enhances employees’ organizational commitment and boosts their sense of self-efficacy. As such, women who experience a more positive diversity environment encounter decreased conflict with both coworkers and managers. Furthermore, they demonstrate elevated job engagement and reduced levels of burnout (28).

The current data showed that experiences of burnout specifically differed in women with respect to their level of education and in men depending on whether they received help at home, were involved in the online learning of their children and satisfied with their professional productivity. Men, while facing challenges similar to women in terms of remote work and blurring boundaries, may also contend with traditional expectations of being the primary breadwinners. Hence, maintaining job performance during economic uncertainty may have alleviated burnout in Lebanese men. Interestingly, men who have taken on increased caregiving responsibilities during the pandemic experienced less burnout than those who did not. This may suggest that these men adjusted to non-traditional roles, succeeded in striking a balance between work and family life and enjoyed parental interactions. Indeed, increased positive emotions in parenting as well, including feelings of closeness to children and gratitude was reported during COVID-19 lockdown (24).

An extensive body of literature has investigated the impact of the COVID-19 pandemic on burnout among healthcare providers (29) and academicians (30) while focusing on gender differences. The current study assessed the impact of the COVID-19 pandemic on burnout among Lebanese men and women, however its findings should be interpreted in light of the peculiar situation of Lebanon, as the country has been struggling for the past 4 years with an unprecedented compounded crisis that has reverberated across all facets of life. In addition to the COVID-19 pandemic, Lebanon’s economic crisis and the 2020 Beirut Port Explosion were reported as the main stressors by both women and men. Therefore, one cannot dissociate the effect of those on the burnout levels that we report herein. Job insecurity and financial stress may add to the burnout experienced by many Lebanese women and men, hence amplifying feelings of helplessness and anxiety. Indeed, several studies have actually examined the mental health difficulties in Lebanon resulting from the aftermath of the compounded crisis, mostly investigating post-traumatic stress disorder, depression, anxiety, stress, somatic symptom disorders, and functional impairment (31–33) but not burnout. To the best of our knowledge, this is the first study that explores the level of burnout and gender-related factors in Lebanese men and women during the pandemic and in light of a multifaceted crisis.

## Limitations

However, the authors acknowledge the following limitations of this study. The data was collected through a snowball sampling method and an online questionnaire, potentially excluding individuals without internet access or limited social media presence. This may restrict the generalizability of the findings. Another notable limitation is the high educational level of our participant cohort, which could have introduced selection bias. Additionally, the data is limited to a cross-sectional design, making it challenging to account for unobservable and time-invariant factors that could significantly influence the gender difference in burnout. Replicating the study results using longitudinal data would be valuable in eliminating potential bias associated with these factors. Furthermore, this study focuses exclusively on workers in Lebanon. While developed countries generally exhibit similar market and institutional conditions, individual cultural and environmental factors can possibly influence the perception of job burnout. Consequently, the study findings may be extrapolated to countries with a similar institutional and social fragility (34). The current study sample of respondents included a small number of healthcare professionals which represent a unique occupational group with an increased risk of burnout. For such group, the authors acknowledge that the Maslach Burnout Inventory – Human Services for Medical Personnel (MBI-HSS (MP)) constitutes a more valid tool to measure burnout in such category of workers but was not the focus of the study.

## Conclusion

This research enriches existing literature by identifying burnout risk factors and informing targeted interventions in Lebanon, with a focus on the gender dimensions of burnout during the pandemic.

Future research should focus on longitudinal studies to understand the long-term effects of crises on burnout and mental health. Qualitative studies exploring personal experiences and cultural influences, along with investigations into the effectiveness of targeted interventions like gender-specific support programs, are critical. Additionally, comparative research across different socio-economic contexts and studies on intersectional factors such as age and caregiving responsibilities can provide deeper insights into burnout prevention and management.

## Data Availability

The original contributions presented in the study are included in the article/supplementary material, further inquiries can be directed to the corresponding author.
